# Models for the Anionic Polymerization of P═C Bonds: Cyclization of *n*‐BuLi‐Initiated MesP═CPh_2_ and Related Phosphaalkenes with H_2_C═CPh_2_

**DOI:** 10.1002/chem.202500389

**Published:** 2025-04-16

**Authors:** Tian Zhang, Kurt F. Hoffmann, Brian O. Patrick, Derek P. Gates

**Affiliations:** ^1^ Department of Chemistry University of British Columbia 2036 Main Mall Vancouver British Columbia V6J 1L4 Canada

**Keywords:** cycloaddition, main group elements, multiple bonds, phosphaalkenes, phosphanes

## Abstract

To model the first propagation step in the anionic polymerization of MesP═CPh_2_ we studied the addition of Li[MesP(Bu)–CPh_2_] (and related species) to nonpolymerizable H_2_C═CPh_2_. Addition proceeds via the *o*‐CH_3_ of the P‐Mes followed by unprecedented cyclization to C_5_P‐rings with release of Li[CHPh_2_]. Further investigation of the aforementioned reaction using phosphaalkenes, RP═CAr_2_ (R ═ Mes, *m*‐Xyl; R′ ═ Ph, 4‐FC_6_H_4_, 4‐MeC_6_H_4_, 4‐MeOC_6_H_4_), resulted in the observation of a relatively long‐lived intermediate in two instances (R ═ Mes, R’ = 4‐MeC_6_H_4_, 4‐MeOC_6_H_4_). For the latter, the intermediate was identified as *n*‐BuP(CH(4‐MeOC_6_H_4_)_2_)[C_6_(4,6‐Me_2_)H_2_–(2‐CH_2_CH_2_CPh_2_Li) by isolation of the oxidized, H^+^‐quenched product. These observations provide intriguing clues into the complex mechanism of polymerization of *P*‐Mes phosphaalkenes and the chiral cyclophosphane products are of interest as ligands for catalytic applications.

## Graphical abstract

Noninnocence of *P*‐Mesityl substituent, cleavage of a P═C bond and cyclization are observed in reactions of five phosphaalkenes. The observed activation of the C─H bond of the Mesityl substituent and subsequent addition to nonpolymerizable H_2_C═CPh_2_ is examined as a potential clue to the polymerization mechanism for *P*‐Mesityl phosphaalkenes.

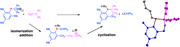

## 1 Introduction

Phosphaalkenes (RP═CR_2_) are compounds with a (3p‐2p)π bond between phosphorus and carbon.^[^ ^1^ ^]^ As formal analogues of alkenes, P═C bonds have become versatile functional groups and building blocks in organophosphorus chemistry. In recent years, P═C bonds have been of growing interest for applications in synthesis, catalysis, polymers, and materials chemistry.^[^ ^2^ ^]^ Although short‐lived HP═CH_2_, HP═CF_2_, and ClP═CH_2_ had been detected spectroscopically,^[^ ^3^ ^]^ the advent of bottleable phosphaalkenes in the mid‐1970′s came with the realization that large substituents could impart kinetic and thermodynamic stabilization to the P═C bond.^[^ ^4^, ^5^ ^]^ Substituents such as *t*‐Bu, Mes, Mes* (Mes* = 2,4,6‐*t*‐Bu_3_C_6_H_2_) permitted rapid initial growth in the chemistry of heavier element multiple bonds and they continue to be widely used in main group multiple bond chemistry.^[^ ^6^ ^]^

**Scheme 1:**
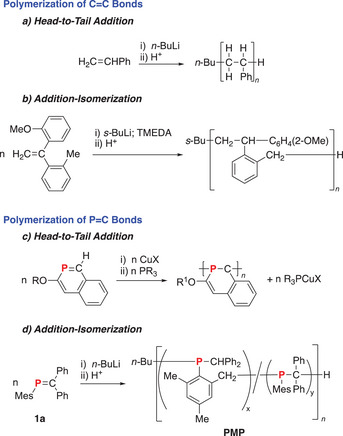
Enchainment pathways for the polymerization of C═C bonds of olefins and P═C bonds of phosphaalkenes showing: a) the most common head‐to‐tail addition polymerization of olefins (for example, the anionic polymerization styrene is shown here); b) the anion‐initiated addition‐isomerization anionic polymerization of a C═C bond; c) the head‐to‐tail addition polymerization of 2‐phosphanaphthalene; d) the anion‐initiated addition‐isomerization polymerization of **1a**.

We have been interested in employing P═C bonds as monomers for the synthesis of novel functional phosphine polymers, poly(methylenephosphine)s (**PMP**s).^[^ ^7^ ^]^ To this end, we developed the first polymerization of MesP═CPh_2_ (**1a**) and related phosphaalkenes.^[^ ^8^, ^9^ ^]^ The elucidation of the mechanism of propagation for this fascinating polymerization reaction has been very challenging due to the difficulty in determining polymer microstructure. In contrast to the simple head‐to‐tail addition mechanism observed for olefins [Scheme 1(a)], model studies have revealed that **1a** appears to follow a primarily addition‐isomerization mechanism when initiated by radicals or anions [Scheme 1(d)].^[^ ^10^, ^11^ ^]^ Specifically, a hydrogen transfer from the o‐Me moiety of the Mes to the −CPh_2_ moiety appears to play a key role in propagation in both the radical‐ and anion‐initiated polymerization. Recently, the first addition‐isomerization mechanism for a C═C bond was proposed for the otherwise nonpolymerizable 1,1‐diphenylethylene derivative, H_2_C═C(*o*‐Tol)(*o*‐MeOC_6_H_4_) [Scheme 1(b)].^[^ ^12^ ^]^ To date, the only regular enchainment of P═C bonds has been observed in the polymerization of monomeric 2‐phosphanaphthalenes mediated by copper(I) halides [Scheme 1(c)].^[^ ^13^ ^]^

Our early molecular model studies of the polymerization of **1a** provided no evidence for involvement of the *o*‐CH_3_ moiety of Mes.^[^ ^14^ ^]^ In all cases, the addition of electrophiles (E^+^) to Li[MesP(Me)─CPh_2_] afforded MesP(Me)─CPh_2_E [E ═ H, Me, P(NEt_2_)_2_, SiMe_3_, SiMe_2_H], consistent with a simple addition mechanism. A later study, found that treatment of Li[ArP(Me)─CPh_2_] (Ar ═ Mes, *m*‐Xyl) with bulky Ph_3_C‐Cl which afforded the C─H activated Ph_3_C─CH_2_(C_6_Me_2_H_2_)P(Me)−CPh_2_H (Ar ═ Mes, *m*‐Xyl), supportive of the addition‐isomerization mechanism. We have also provided evidence that living anionic [**PMP**]‐MesP─CPh_2_^−^ initiates the polymerization of methyl methacrylate via the ─CPh_2_ moiety, suggesting a normal addition at the switching group.^[^ ^15^ ^]^ To date, molecular model studies have not demonstrated that the living phosphaalkene anion can add to an E═E′ bond via the *o*‐CH_3_ moiety. This is critical to developing a model for the propagation step in the anionic polymerization of **1a**.

## 2 Results and Discussion

**Scheme 2:**
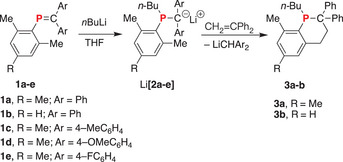
Intramolecular cyclization of phosphaalkenes (**1a‐e**) to phosphacyclohexenes (**3a‐b**) from the stepwise addition of *n*‐BuLi (1 equiv) and H_2_C═CPh_2_ (1 equiv).

Herein, we provide the first structural evidence for addition of phosphaalkene anion, Li[**2a**], to a C═C double bond via the *o*‐CH_3_ moiety rather than the −CPh_2_ anion. This reaction is general for a variety of phosphaalkenes (**1a‐e**) and provides an important molecular model for the propagation step in the anionic polymerization phosphaalkenes. In addition, this isomerization‐addition step is followed by an unprecedented cyclization to afford novel C_5_P heterocycles (**3a‐b**) and may provide important clues to the unusual polymerization mechanism of P═C bonds (Scheme 2).

To model the propagation step in the anionic polymerization of phosphaalkene (**1a‐e**), H_2_C═CPh_2_ was chosen since it is well‐known as a nonpolymerizable monomer yet it is susceptible to anion addition. To this end, a solution of *n*‐BuLi in hexanes was added to a THF solution of **1a** (1 equiv). The red reaction mixture was analyzed by ^31^P NMR spectroscopy to reveal that the signal assigned to **1a** (δ = 233.5) was no longer present and only a singlet resonance was detected at −26.5 ppm which was assigned to Li[**2a**] by comparison to our previous work.^[^ ^14^ ^]^ Subsequently, a solution of H_2_C═CPh_2_ (1 equiv) in THF was added. Although no color change was observed, analysis of the reaction mixture by ^31^P NMR spectroscopy revealed that signal assigned to Li[**2a**] (δ = −26.5) had been fully consumed and was replaced by a singlet resonance at −30.7 ppm. The addition of excess methanol to the deep‐red reaction solution resulted in the formation of a pale‐yellow solution.

**Figure 1:**
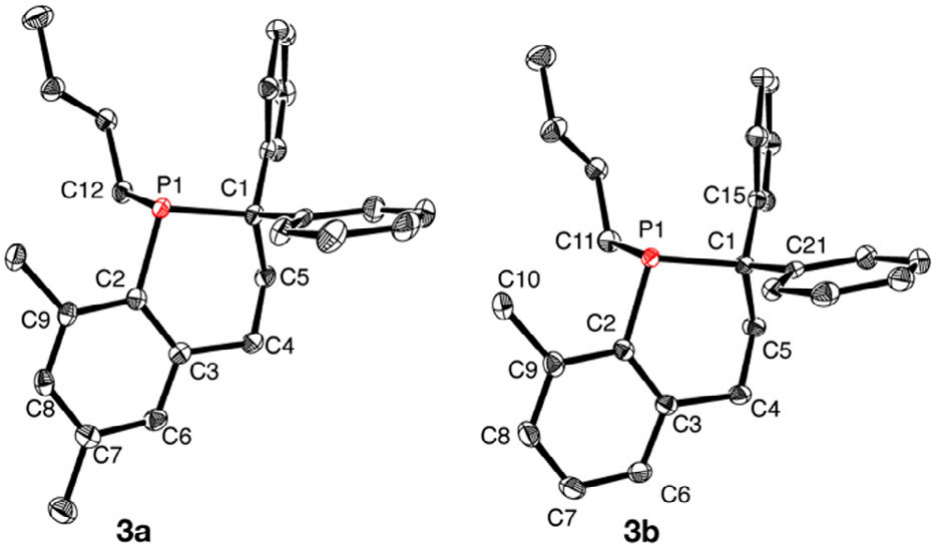
Molecular structure of **3a** and **3b** (50% probability ellipsoids). For **3b**, one of the two independent molecules in the asymmetric unit is shown (Molecule A). Hydrogen atoms are omitted for clarity. Selected bond lengths (Å) for **3a**: P1─C1 1.892(2), P1─C2 1.841(2), P1─C12 1.864(2). Selected bond lengths (Å) for **3b**: Molecule A: P1─C1 1.890(2), P1─C2 1.849(2), P1─C11 1.869(2); Molecule B: P1─C1 1.891(2); P1─C2 1.844(2); P1─C11 1.867(2).

After work‐up, the crude product was isolated as a pale‐yellow oil, again showing a singlet ^31^P NMR resonance at −30.7 ppm. Crystals suitable for X‐ray analysis were grown by cooling a saturated solution of the crude product in Et_2_O (−35 °C). Remarkably, the molecular structure revealed the product to be phosphacyclohexene **3a** (Figure 1). The metrical parameters will be discussed below. Given the popularity of the Mes substituent is widely used in P‐chemistry, there are strikingly few examples of reactions at the *o*‐CH_3_ moiety in the absence of a metal.^[^ ^16^ ^]^ Although C_5_P‐heterocycles are well‐established,^[^ ^17^ ^]^ six‐membered benzo‐fused phosphine rings, the so‐called 1,2,3,4‐tetrahydrophosphinolines, similar to **3a**, are quite rare. Phosphine analogues (5,6‐C_6_H_4_)(CH_2_)_3_(PR) (R ═ Et,^[^ ^18^, ^19^ ^]^ Cl^[^ ^19^ ^]^) and phosphajulolidine‐derivative P[CH_2_CH_2_CMe_2_]_2_(4_C_,5_P_,6_C_‐C_6_H_3_)^[^ ^20^, ^21^ ^]^ are known but have not been structurally characterized. In addition, a small number of related six‐membered cyclophosphahexenes have been structurally characterized where the P atom is further alkylated, oxidized, or bound to borane.^[^ ^22^ ^]^ Phosphacyclohexene **3a** was fully characterized using a combination of ^1^H, ^31^P, ^13^C{^1^H} and 2D NMR spectroscopy, FDMS, and elemental analysis. Density functional theory (DFT) [B3LYP‐D3(BJ)/def2‐TZVPP] was used to evaluate the energetics of the reaction using THF as implicit solvent model (CPCM, ε = 7.25). The computations suggest that products **3a** and [CHPh_2_]^–^ were thermodynamically preferred over [**2a**]^−^ and H_2_C═CPh_2_ [*ΔG*° = −13.44 kcal mol^−1^].

To explore the generality of this intramolecular cyclization and gain insight into the reaction mechanism, several additional phosphaalkenes were examined. Namely, THF solutions of **1b‐e** were treated with *n*‐BuLi (1 equiv) in hexane. In all cases, the initially colorless solution of **1b‐e** immediately turned deep red upon addition of *n*‐BuLi. Analysis of an aliquot removed from the reaction mixture by ^31^P NMR spectroscopy revealed that the signals assigned to each phosphaalkene [δ (range) = 217.7–233.8] had been replaced by a new upfield‐shifted resonance assigned to Li[**2b‐e**] {δ = −25.1, Li[**2b**]; −25.6, Li[**2c**]; −26.9, Li[**2d**]; −24.6, Li[**2e**]}. Subsequently, H_2_C═CPh_2_ (1 equiv) was added and each dark‐red reaction mixture was stirred for one day and visually appeared deeper in color, particularly in the case of Li[**2e**]. Analysis by ^31^P NMR spectroscopy revealed that the signal assigned to Li[**2b‐e**] was no longer present and a singlet resonance was detected at either −30.5 {for reaction of [**2b**]^−^} or −30.7 ppm {for reaction of [**2c**]^−^, [**2d**]^−^, and [**2e**]^−^}. The latter signal corresponded exactly to that previously assigned to **3a** suggesting that the same cyclization had occurred. In these reactions, the leaving groups were presumably: [CH(C_6_H_4_Me)_2_]^−^, [CH(C_6_H_4_OMe)_2_]^−^, and [CH(C_6_H_4_F)_2_]^–^, respectively. Each reaction was quenched by the addition of MeOH (1 drop) and the identity of the product as **3a** was confirmed by NMR spectroscopy and X‐ray crystallography. In each case, sufficient data were collected to both determine the unit cell and to establish the connectivity of the molecule making up the crystal. All results are consistent with each product being **3a**. In the case of the reaction of [**2b**]^−^, the suspected formulation of the product as **3b** was confirmed by X‐ray crystallographic analysis. The molecular structure is shown in Figure 1 and again confirms the unique benzo‐fused C_5_P‐heterocyclic structure. In addition, the product was fully characterized using ^1^H, ^31^P, ^13^C{^1^H} and 2D NMR spectroscopy, FDMS, and elemental analysis.

The metrical parameters of **3a** and **3b** do not differ considerably. Both heterocycles adopt half‐chair structures with P1, C2, C3, and C4 being nearly co‐planar and C1 being only slightly out of this best‐plane [distance to plane (Å) = 0.115(3), **3a**; 0.153(3), **3b**], and C5 is bent considerably out of plane [distance to plane (Å) = −0.622(4), **3a**; −0.578(4), **3b**]. The P─C bonds [avg. 1.867(2) Å] are at the long end of the typical range of P–C single bonds [avg. (Å): P─C_sp_3 = 1.855(19); P─C_Ar_ = 1.836(10)]^[^ ^23^ ^]^ with the hindered P1─C1 bonds [avg. 1.891(2) Å] being longer than P1─C2 [avg. 1.845(2) Å]. The angles within the phosphacyclohexene ring are all slightly more obtuse than expected, suggesting possible ring‐strain. For instance, the C1─P1─C2 angle [102.42(8)°, **3a**; 102.5(1)°, **3b**] is slightly larger than that found in acyclic MesP(Me)CHPh_2_ [100.18(6)°].^[^ ^14^ ^]^ In addition, the internal ring angles at the sp^3^ carbon atoms [P1─C1─C5_avg_ = 110.4(1)°; C1─C5─C4_avg_ = 113.0(2)°; C5─C4─C3_avg_ = 115.3(2)°] are greater than those expected for idealized sp^3^ carbon. The internal ring angles at the sp^2^ carbon atoms [P1─C2─C3_avg_ = 123.8(2)°; C2─C3─C4_avg_ = 123.1(1)°].

**Figure 2:**
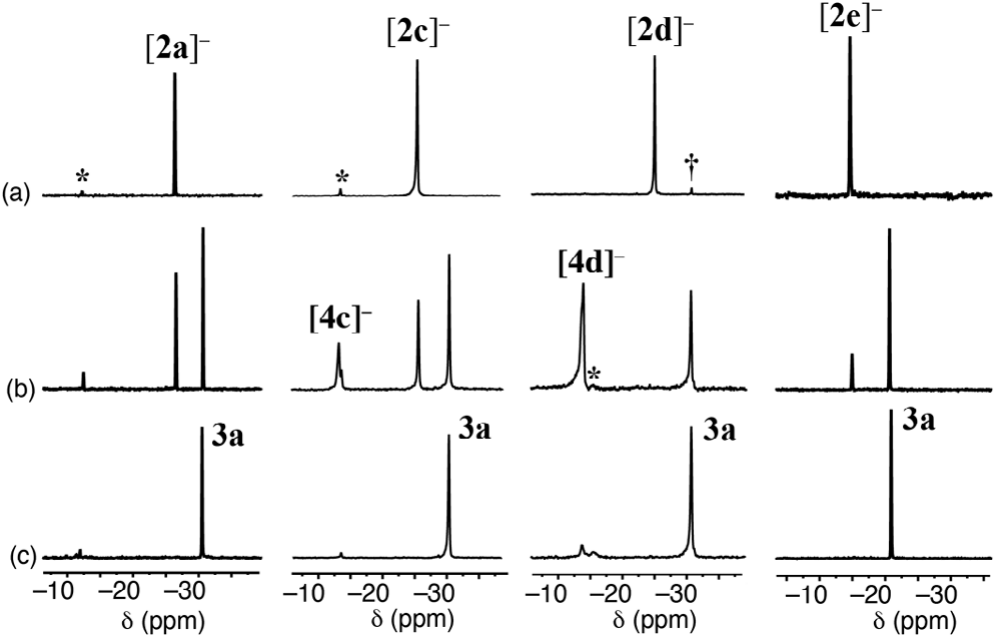
^31^P NMR (162 MHz, THF, 198 K) spectra of reaction progress of Li[**2a,c,d,e**] with H_2_C═CPh_2_ (1 equiv) to afford cyclization product **3a**: a) solutions of Li[**2]** in THF; b) immediately after addition of H_2_C═CPh_2_ (1 equiv); c) one day after addition of H_2_C═CPh_2_ (1 equiv). * denotes signals assigned to quenched product, ArP(Bu)─CHR_2_ [**2a,c,d,e**]─H, presumably formed from traces protic impurities. † denotes unassigned.

**Scheme 3:**
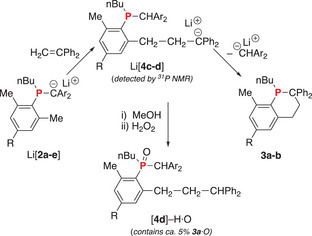
Proposed mechanism of reaction for Li[2**a**‐**e**] with H_2_C═CPh_2_ to afford isolable products, 3**a**‐**b**. Intermediates have been detected by ^31^P NMR spectroscopy in two cases and have been assigned to Li[4**c**‐**d**] on the basis of isolated and characterized [**4d**]─H·O.

With the identity of cyclized products **3a‐b** confirmed, it remained to evaluate the mechanism of formation from Li[**2a‐e**] and H_2_C═CPh_2_. Thus, solutions of Li[**2a‐e**] in THF were prepared from **1a‐e** and *n*‐BuLi under rigorously anhydrous conditions. ^31^P NMR spectra of selected examples are shown in Figure 2(a). To each stirred solution was rapidly added a solution of H_2_C═CPh_2_ (1 equiv) in THF and an aliquot was immediately transferred to an NMR tube. The ^31^P NMR spectra are shown in Figure 2(b). As expected, all reactions show that the signals assigned to [**2a‐e**]^−^ decreased in intensity as the signals assigned to **3a‐b** increased. Particularly striking are the reactions involving [**2c**]^−^ and [**2d**]^−^. In these cases, a signal attributed to an intermediate species was observed at −13.5 and −13.9 ppm, respectively. Each broad signal displayed a shoulder of lower intensity (c.a. 2:3), shoulder slightly downfield (ca. Δδ ≈ 0.2) (*vide infra*). This signal, along with that assigned to starting [**2c‐d**]^−^, was ultimately fully consumed as shown in Figure 2(c). We tentatively assign this signal to intermediate species [**4c**]^−^ and [**4d**]^−^ and propose the following pathway for the formation of **3a‐b** from Li[**2a‐e**] (Scheme 3). We note that these intermediates are observed with the poorer leaving groups and not observed with the better leaving groups {(worst) [CH(4‐MeOC_6_H_4_)_2_]^−^ < [CH(4‐MeC_6_H_4_)_2_]^−^ < [CHPh_2_]^−^ < [CH(4‐FC_6_H_4_)_2_]^−^ (best)}.

In an effort to confirm the identity of the intermediate tentatively assigned as Li[**4d**], the reaction mixture was protonated to obtain a neutral product. Thus, a solution containing Li[**4d**] (major), **3a** (minor), and [**2d**]‐H (trace), nearly identical to that shown in Figure 2(b), was generated and immediately treated with MeOH (1 drop). Analysis of this reaction mixture by ^31^P NMR spectroscopy revealed signals assigned to [**4d**]─H [δ = −13.6 (sh), −14.0; overall 68%; intensity ratio ≈ 1:3], along with signals assigned to **3a** (δ = −30.7; 27%;), and [**2d**]−H (δ = −14.4; 5%). The percentages presented represent the ratio of signal areas in the ^31^P NMR spectrum and may not be accurate to the exact product ratio due to differences in ^31^P relaxation times. Efforts to separate the products were unsuccessful due to their air sensitivity. Thus, the crude product mixture was isolated, dissolved in CH_2_Cl_2_, and oxidized with H_2_O_2_. After work‐up, the ^31^P NMR spectrum of the crude product mixture in CH_2_Cl_2_ showed resonances assigned to [**4d**]–H·O [δ = 47.8 (sh), 47.3; intensity ratio ≈ 1:5; 53%], **3a**·O (δ = 38.3; 44%), and [**2d**]─H·O (δ = 24.2; 3%]. The presence of shoulders for the signals assigned to [**4d**]─H and [**4d**]─H·O is attributed to diastereomers resulting from the P‐stereocenter and atropisomerism arising from restricted rotation of the former P‐Mes substituent, now containing a long CH_2_CH_2_CHPh_2_ side chain. Column chromatography on silica using CH_2_Cl_2_ as eluant afforded [**4d**]─H·O with a small amount of **3a**·O (ca. 5%) according to ^31^P NMR spectroscopic analysis. In addition, no Ph_2_CH_2_ was observed by ^1^H NMR spectroscopy. Although crystals of [**4d**]─H·O suitable for X‐ray diffraction were not obtained, the formulation of [**4d**]─H·O was supported by the assignment of key ^1^H ^13^C{H} NMR signals using HSQC and HMBC NMR spectroscopic experiments. Full assignment was very difficult due to the presumed diastereomeric nature of [**4d**]─H·O described above. Moreover, FDMS provided evidence for the molecular ion [*m*/*z* (%) = 614.54 (63), 615.54 (30), 616.54 (7)].

The observation of species such as Li[**4c‐d**] and **3a‐b** could be valuable in the elucidation of the addition‐isomerization polymerization of **1a** and related derivatives. DFT calculations using the solvent model described above suggest that the formation of putative intermediate [**4a**]^−^ from [**2a**]^−^ and H_2_C═CH_2_ is endoergic [*ΔG*° = 16.10 kcal mol^−1^]. The subsequent cyclization of [**4a**]^−^ to afford **3a** and [CHPh_2_]^−^ was exoergic [*ΔG*° = −29.55 kcal mol^−1^]. Given that H_2_C═CPh_2_ cannot be polymerized, the cyclization to **3a** and LiCHPh_2_ illustrates an alternate, although unexpected, fate for Li[**4a**]. In contrast, treatment of a THF solution of Li[**2a**] with polymerizable **1a** (1 equiv) does not form a single product as determined by ^31^P NMR spectroscopy. In fact, Li[**2a**] initiates polymerization in the presence of excess **1a**. We have previously examined the oligomers of **1a** initiated with *n*‐BuLi (via Li[**2a**]) and have hypothesized that some oligomeric species detected in MALDI‐TOF MS could be consistent with backbiting reactions.^[^ ^24^ ^]^ On the basis of the present results with **1a**‐surrogate, H_2_C═CPh_2_, it begs the question of whether cyclic analogues of **3a‐b** (i.e., C_4_P_2_ cycles) may be involved in the novel anion‐initiated addition‐isomerization polymerization of P‐Mes phosphaalkenes, **1a‐e**, to **PMP**s. Moreover, the effect of leaving group may offer opportunities to finely tune the open‐chain versus cyclic structures. These studies are underway and shall be reported separately.

## 3 Conclusion

In summary, the addition of anion‐initiated **1a‐e** to H_2_C═CPh_2_ provides molecular models of the first propagation step in the polymerization of P‐Mes and related phosphaalkenes. Consistent with the hypothesized mechanism of polymerization, the activation of the *o*‐CH_3_ moiety of the P‐Mes (or *m*‐Xyl) substituent was observed in all cases. This isomerization‐addition step was followed by a quantitative, although surprising, intramolecular cyclization which provides an important clue to the, as yet not fully understood, polymerization mechanism. Moreover, the new bulky chiral phosphines reported are of interest as ligands for catalytic applications.

## Supporting Information

The authors have cited additional references within the Supporting Information.^[^ ^25^, ^26^, ^27^, ^28^, ^29^, ^30^, ^31^, ^32^, ^33^, ^34^, ^35^, ^36^ ^]^ Experimental procedures, characterization data, NMR spectra, are given in the supporting information. Deposition Number(s) 2 355 932 (**3a**), 2 429 705 (**3b**) contain(s) the supplementary crystallographic data for this paper. These data are provided free of charge by the joint Cambridge Crystallographic Data Centre and Fachinformationszentrum Karlsruhe Access Structures service.

## Conflict of Interests

The authors declare no conflict of interests.

## Supporting information



Supporting Information

## Data Availability

The data that support the findings of this study are available in the supplementary material of this article.
