# Supplementary material for: Models for the Anionic Polymerization of P═C Bonds: Cyclization of n‐BuLi‐Initiated MesP═CPh2 and Related Phosphaalkenes with H2C═CPh2
Source: Chemistry. 2025 Apr 16;31(25):e202500389. doi: 10.1002/chem.202500389 (PMC12057597; doi:10.1002/chem.202500389)
Supplement: Supplementary file 1 — Supporting Information [file CHEM-31-e202500389-s001.pdf]

Supporting Information

Models for the Anionic Polymerization of P=C bonds: Cyclization of *n*-BuLi-Initiated MesP=CPh_2 and Related Phosphaalkenes with H_2C=CPh_2

Tian Zhang, Kurt F. Hoffmann, Brian O. Patrick, and Derek P. Gates

Department of Chemistry, University of British Columbia, 2036 Main Mall, Vancouver, British Columbia, Canada, V6J 1L4.

dgates@chem.ubc.ca

Experimental Section:

General Procedures. All manipulations of air- and/or moisture-sensitive compounds were conducted using standard Schlenk or glovebox techniques. Tetrahydrofuran was dried over sodium/benzophenone ketyl and distilled prior to use. Hexanes, dichloromethane and diethyl ether were degassed and passed through an activated alumina column prior to use. ^{1}H,$^{13}C\{^{1}H\}$, and ^{31}P NMR spectra were measured at room temperature on a Bruker AVANCEIII HD 400 MHz spectrometer. All NMR spectra were recorded at room temperature unless otherwise specified. Chemical shifts of ^{1}H NMR spectra were expressed in parts per million and referenced to residual protonated solvent in $CDCl_3$ (δ = 7.26)[25] Chemical shifts of $^{13}C\{^{1}H\}$ NMR spectra were expressed in parts per million and references to solvent [$CDCl_3$: δ = 77.16].[26] Chemical shifts of ^{31}P NMR spectra were expressed in parts per million and references to 85% H_3PO_4 as an external standard (δ = 0).

Synthesis of phosphacyclohexene (3a):

Path A (from 1a): To a solution of MesP=CPh_2 (**1a**) (0.358 g, 1.13 mmol) in THF (3 ml) was added a solution of *n*-BuLi in hexane (0.707 ml, 1.6 M, 1.13 mmol) at room temperature. The mixture turned dark red upon addition and was stirred for 15 min. The reaction was monitored by ^{31}P NMR (162 MHz) spectroscopy, affording a singlet resonance at –26.5 ppm assigned to Li[**2a**]. Subsequently, a solution of diphenylethylene (0.204 g, 1.13 mmol) in THF (2ml) was added rapidly. At this time, an aliquot was removed from the reaction mixture and analyzed by ^{31}P NMR spectroscopy, which showed two resonances (δ = –26.5 and δ = –30.7). The resonance at δ = –26.5 was assigned to MesP(Bu)-C(Li)Ph_2. The new resonance at δ = –30.7 was assigned to **3a**. After the mixture was stirred for one day, ^{31}P NMR analysis showed complete conversion to a singlet resonance (δ = –30.7). To the stirred carbanion model compound in THF was added dropwise degassed MeOH until the reaction mixture turned pale yellow (several drops). Then the solvent was removed in vacuo at 60 °C and the yellow residue was dissolved in hexane and filtered through a silica plug. A yellow fraction was collected and the solvent was removed in vacuo to afford a yellow solid. A saturated solution of the crude product in diethyl ether was cooled to–35 °C to afford crystals of **3a** (0.285 g, 65.4%).

Path B (from 1c): To a solution of MesP=C(4-MeC_6H_4)$_2$ (**1c**) (0.463, 1.34 mmol) in THF (4 ml was added a solution of *n*-BuLi in hexane (0.841 ml, 1.6 M, 1.34 mmol) at room temperature. The

mixture turned dark red upon addition and was stirred for 15 min. The reaction was monitored by ^{31}P NMR (162 MHz) spectroscopy, affording a single signal at -25.6 ppm assigned to Li[**2c**]. Subsequently, a solution of diphenylethylene (0.243 g, 1.34 mmol) in THF (2 ml) was added rapidly. At this time, an aliquot was removed from the reaction mixture and analyzed by ^{31}P NMR spectroscopy, which showed three resonances (δ = –13.2, –25.6 and δ = –30.7). The resonance at δ = –25.6 was assigned to MesP(Bu)-C(Li)(4-MePh)$_2$. The resonance at δ = –13.2 was an intermediate during the reaction. The new resonance at δ = –30.7 was assigned to the **3a**. After the mixture was stirred for one day, ^{31}P NMR analysis showed complete conversion to a singlet resonance (δ = –30.7). To the stirred carbanion model compound in THF was added dropwise degassed MeOH until the reaction mixture turned pale yellow (several drops). Then the solvent was removed in vacuo at 60 °C and the yellow residue was dissolved in hexane and filtered through a silica plug. A yellow fraction was collected and the solvent was removed in vacuo to afford a yellow solid. A saturated solution of the crude product in diethyl ether was cooled to–35 °C to afford crystals of **3a** (0.326 g, 64.9%).

Path C (from 1d): To a solution of MesP=C(4-OMeC$_6$H$_4$)$_2$ (**1d**) (0.483 g, 1.28 mmol) in THF (4 ml) was added a solution of *n*-BuLi in hexane (0.803 ml, 1.6 M, 1.28 mmol) at room temperature. The mixture turned dark red upon addition and was stirred for 15 min. The reaction was monitored by ^{31}P NMR (162 MHz) spectroscopy, affording a single signal at –26.9 ppm assigned to Li[**2d**]. Subsequently, a solution of diphenylethylene (0.231 g, 1.28 mmol) in THF (2 ml) was added rapidly. At this time, an aliquot was removed from the reaction mixture and analyzed by ^{31}P NMR spectroscopy, which showed two resonances (δ = –13.6 and δ = –30.7). The resonance at δ = –13.6 was an intermediate during the reaction. The resonance at δ = –30.7 was assigned to **3a**. After the mixture was stirred for one day, ^{31}P NMR analysis showed complete conversion to a singlet resonance (δ = –30.7). To the stirred carbanion model compound in THF was added dropwise degassed MeOH until the reaction mixture turned pale yellow (several drops). Then the solvent was removed in vacuo at 60 °C and the yellow residue was dissolved in hexane and filtered through a silica plug. A yellow fraction was collected and the solvent was removed in vacuo to afford a yellow solid. A saturated solution of the crude product in diethyl ether was cooled to–35 °C to afford crystals of **3a** (0.380 g, 49.5%).

Path D (from 1e): To a solution of MesP=C(4-FC$_6$H$_4$)$_2$ (**1e**) (0.302, 0.86 mmol) in THF (3 ml) was added a solution of *n*-BuLi in hexane (0.536 ml, 1.6 M, 0.86 mmol) at room temperature. The mixture turned dark red upon addition and was stirred for 15 min. The reaction was monitored by ^{31}P NMR (162 MHz) spectroscopy, affording a single signal at –24.6 ppm assigned to Li[**2e**]. Subsequently, a solution of diphenylethylene (0.154 g, 0.86 mmol) in THF was added. At this time, an aliquot was removed from the reaction mixture and analyzed by ^{31}P NMR spectroscopy, which showed two resonances (δ = –24.6 and δ = –30.7). The resonance at δ = –24.6 was assigned to Li[**2e**]. The new resonance at δ = –30.7 was assigned to **3a**. After the mixture was stirred for one day, ^{31}P NMR analysis showed complete conversion to a singlet resonance (δ = –30.7). To the stirred carbanion model compound in THF was added dropwise degassed MeOH until the reaction mixture turned pale yellow (several drops). Then the solvent was removed in vacuo at 60 °C and the yellow residue was dissolved in hexane and filtered through a silica plug. A yellow fraction was collected and the solvent was removed in vacuo to afford a yellow solid. A saturated solution of the crude product in diethyl ether was cooled to–35 °C to afford crystals of **3a** (0.198, 59.8%).

Data for 3a. ^{31}P NMR (162 MHz): δ −30.7 (s). ^{1}H NMR (400 MHz, CDCl$_3$) δ 7.34 – 7.12 (m, 10H, Ar), 6.92 (s, 1H, *m*-Mes), 6.72 (s, 1H, *m*-Mes), 2.83 (m, 1H, *o*-C*H*H-Mes), 2.70 (m, 1H, P–CPh$_2$–C*H*H–), 2.56 (s, 3H, *o*-CH$_3$-Mes), 2.41 (m, 1H, P–CPh$_2$–CH*H*–), 2.26 (m, 1H, *o*-CH*H*-Mes),

2.28 (s, 3H, *p*-CH_3-Mes), 1.45 (m, 1H, P–C*H*H), 1.17 (m, 1H, P–CH_2–CH*H*), 1.09 (m, 1H, P–CH_2–CH_2–C*H*H), 0.96 (m, 1H, P–CH_2–CH_2–CH*H*), 0.86 (m, 1H, P–C*H*H), 0.61 (t, $^3J_{HH}$ = 7 Hz, 3H, P–$(CH_2)_3$–CH_3), 0.08 (m, 1H, P–CH*H*). ^{13}C NMR (101 MHz, $CDCl_3$) δ 147.66 (d, *J* = 16 Hz, *ipso*-Ar), 146.94 (d, *J* = 6 Hz, *ipso*-Ar), 141.55 (d, *J* = 24 Hz, *o*-Mes), 140.62 (s, *o*-CH_2–Mes), 137.51 (s, *p*-Mes) 131.11 (d, *J* = 19 Hz, *ipso*-Mes), 129.55 (d, *J* = 4.0 Hz, *m*-Mes), 129.37–128.62 (Aromatic carbon), 126.00 (d, J = 14 Hz, *m*-Mes), 46.82 (d, *J* = 12 Hz, P–C), 30.26 (d, $^1J_{PC}$ = 21 Hz, P–*C*H_2), 30.01 (d, $^2J_{PC}$ = 2 Hz, P–CPh_2–*C*H_2), 29.70 (s, *o*-Mes–*C*H_2), 28.62 (d, $^2J_{PC}$ = 18.0 Hz, P–CH_2–*C*H_2), 24.69 (d, $^3J_{PC}$ = 14 Hz, P–CH_2–CH_2–*C*H_2), 21.55 (d, $^2J_{PC}$ = 24 Hz, *o*–*C*H_3–Mes), 21.48 (s, *p*–CH_3–Mes), 13.97 (s, P–$(CH_2)_3$–*C*H_3). Anal. Calcd for **3a**: C, 83.9; H, 8.08. Found: C, 84.06; H, 8.05. MS (FD, positive mode): $[M]^+$ 386.20 (77), 387.21(23)

Synthesis of Phosphacyclohexene (3b):

To a solution of XylP=CPh_2 (**1b**) (0.315 g, 1.04 mmol) in THF (3 ml) was added a solution of *n*-BuLi in hexane (0.652 ml, 1.6 M, 1.04 mmol) at room temperature. The mixture turned dark red upon addition and was stirred for 15 min. The reaction was monitored by ^{31}P NMR (162 MHz) spectroscopy, affording a single signal at -25.1 ppm. Subsequently, a solution of diphenylethylene (0.188g, 1.04 mmol) in THF (2 ml) was added rapidly. At this time, an aliquot was removed from the reaction mixture and analyzed by ^{31}P NMR spectroscopy, which showed two resonances (δ = –25.1 and δ = –30.5). The resonance at δ = –25.1 was assigned to Li[**2b**]. The new resonance at δ = –30.5 was assigned to **3b**. After the mixture was stirred for one day, ^{31}P NMR analysis showed complete conversion to a singlet resonance (δ = –30.5). To the stirred carbanion model compound in THF was added dropwise degassed MeOH until the reaction mixture turned pale yellow (several drops). Then the solvent was removed in vacuo at 60 °C and the yellow residue was dissolved in hexane and filtered through a silica plug. A yellow fraction was collected and the solvent was removed in vacuo to afford a yellow solid. A concentrated solution of the crude product in diethyl ether was cooled to–35 °C affording crystals of **3b** (0.192 g, 49.5%). ^{31}P NMR (162 MHz, $CDCl_3$) δ -31.0. ^{1}H NMR (400 MHz, $CDCl_3$) δ 7.35 – 6.87 (aromatic region), 2.83 (br m, 1H, *o*-C*H*H-Mes),2.68 (br m, 1H, P–CPh_2–C*H*H–), 2.59 (s, 3H, *o*-CH_3–Xyl), 2.41 (br m, 1H, P–CPh_2–CH*H*), 2.29 (m br, 1H, *o*-CH*H*-Mes), 1.44 (br m, 1H, P–C*H*H), 1.21 (br m, 1H, P–CH_2–C*H*H), 1.10 (br m, 1H, P–CH_2–CH*H*), 0.97 (br m, 1H, P–$(CH_2)_2$–C*H*H), 0.87 (br m, 1H, P–$(CH_2)_2$–CH*H*), 0.59 (t, $^3J_{HH}$ = 7.2 Hz, 3H, P–$(CH_2)_3$–CH_3), 0.08 (m, 1H, P–CH*H*). ^{13}C NMR (101 MHz, $CDCl_3$) δ 147.61 (d, *J* = 16.1 Hz, *ipso*-Ar), 146.81 (d, *J* = 5.9 Hz, *ipso*-Ar), 141.61 (d, *J* = 23.5 Hz, *o*-Xyl), 140.66 (s, *o*–Xyl), 135.65 (d, *J* = 20.5 Hz, *ipso*-Xyl), 129.35-126.50 (aromatic region), 46.84 (d, $^1J_{PC}$ = 12 Hz, P–C), 30.32 (d, *J* = 21.6 Hz, P–*C*H_2), 29.96 (d, $^2J_{PC}$ = 3 Hz, P–CPh_2–*C*H_2), 29.26 (s. *o*-Mes–*C*H_2), 28.43 (d, $^2J_{PC}$ = 18.0 Hz, P–CH_2–*C*H_2), 24.68 (d, $^2J_{PC}$ = 14 Hz, P–$(CH_2)_2$–*C*H_2), 21.67 (d, $^3J_{PC}$ = 24 Hz, *o*-*C*H_3), 13.98 (s, *n*Bu-CH_3). Anal. Calcd for Model compound: C, 83.84; H, 7.85. Found: C, 84.38; H, 7.70. MS (FD, positive mode): $[M]^+$ 372.33 (75), 373.33 (22), 374.34 (3)

Isolation of Intermediate, [4d]–H·O

To a solution of MesP=C(4-OMeC_6H_4)$_2$ (**1d**) (0.297 g, 0.79 mmol) in THF (4 ml) was added a solution of *n*-BuLi in hexane (0.493 ml, 1.6 M, 0.79 mmol) at room temperature. The mixture turned dark red upon addition and was stirred for 15 min. Analysis of an aliquot removed from the reaction mixture by ^{31}P NMR (162 MHz) spectroscopy revealed major signal at –26.9 ppm assigned to Li[**2d**]. After returning the NMR aliquot to the reaction mixture, a solution of diphenylethylene (0.231 g, 0.79 mmol) in THF (2 ml) was rapidly added. An aliquot was removed

from the reaction mixture and analyzed by ^{31}P NMR spectroscopy, which showed resonances at –13.6 assigned to isomers of Li[**4d**], and –30.7 ppm assigned to **3a**. Immediately following the NMR measurement, MeOH (1 drop) was added to the reaction mixture. ^{31}P NMR analysis showed signals at –14.0 ppm with a shoulder at –13.6 ppm along with signals δ = –30.7, and δ = –14.4. The crude product mixture was isolated, dissolved in CH_2Cl_2 and oxidized with H_2O_2. The oxidized mixture was extracted with CH_2Cl_2 three times in air. The organic layer was dried with $MgSO_4$ and the solvent removed in vacuo leaving a colorless solid. The crude product was purified by column chromatography on silica gel using hexane to remove Ph_2CH_2, followed by a second column chromatography on silica gel with CH_2Cl_2. The desired product was isolated with ca. 5% **3a**·O (0.130 g, 26.7%). Data for [**4d**]–H·O: ^{31}P NMR (162 MHz, $CDCl_3$) δ 47.2. ^{1}H NMR (400 MHz, $CDCl_3$) δ 7.83-6.68 20H, aromatic region, 4.42 (d, 1H, –C*H*(4-OMePh)$_2$), 4.07 (s, 1H, –C*H*Ph_2) 3.81 (s, 3H, *para*-C*H*$_3$), 3.59 (s, 2H, Mes-C*H*$_2$), 2.84 (s, 2H, C*H*$_2$-CHPh_2), 2.25 (s, 3H, *ortho*-CH_3), 1.99 (s, 2H, P-C*H*$_2$), 1.53 (q, J = 3.6 Hz, 2H, C*H*$_2$–CH_3), 1.22 (t, J = 5.4 Hz, 2H, C*H*$_2$–CH_2–CH_3), 0.76 (t, J = 7.2 Hz, 3H, CH_2–C*H*$_3$). ^{13}C NMR (101 MHz, $CDCl_3$) δ 158.88-113.84 (aromatic region), 55.65 (s, *para*-*C*H_3), 55.43 (s, Mes-*C*H_2), 53.79 (d, J = 60.2 Hz, –*C*HPh_2), 51.91 (–*C*H(4-OMePh)$_2$, 33.91 (*C*H_2-CHPh_2), 32.02 (d, J = 65.7 Hz, P-*C*H_2), 24.62 (*C*H_2–CH_3), 24.39 (*C*H_2–CH_2–CH_3), 21.37 (*ortho*-*C*H_3), 14.05 (CH_2–*C*H_3). MS (FD, positive mode): *m/z* $[M]^+$ = 614.54 (63), 615.54 (30), 616.54 (7).

General Procedure for ^{31}P NMR monitoring reactions

To a solution of MesP=CPh_2 (**1a**) (0.411 g, 1.29 mmol) in THF (4 ml) was added a solution of *n*-BuLi in hexane (0.812 ml, 1.6 M, 1.29 mmol) at room temperature. The mixture turned dark red upon addition and was stirred for 15 min. The reaction was monitored by ^{31}P NMR (162 MHz) spectroscopy, affording a singlet resonance at –26.4 ppm assigned to Li[**2a**]. And then the NMR sample was put back to the reaction mixture. To stirred solution was rapidly added a solution of H_2C=CPh_2 (0.243 g, 1.29 mmol) in THF and an aliquot was immediately transferred to an NMR tube and the reaction mixture and analyzed by ^{31}P NMR spectroscopy, which showed two resonances (δ = –26.5 and δ = –30.7). The resonance at δ = –26.5 was assigned to MesP(Bu)-C(Li)Ph_2. The new resonance at δ = –30.7 was assigned to model compound. After the mixture was stirred for one day. To the stirred carbanion model compound in THF was added dropwise degassed MeOH until the reaction mixture turned pale yellow (several drops). Then the solvent was removed in vacuo at 60 °C and the yellow residue was dissolved in hexane and filtered through a silica plug. A yellow fraction was collected and the solvent was removed in vacuo to afford a yellow solid. A saturated solution of the crude product in diethyl ether was cooled to –35 °C to afford crystals of **3a** (0.326 g, 64.9%).

X-ray crystallographic data

All single crystals were immersed in oil and mounted on a mylar loop. Data were collected on a Bruker X8 APEX II diffractometer with Mo Kα radiation, integrated using the Bruker SAINT software package,[27] and corrected for absorption effects using SADABS. All structures were solved by direct methods and subsequent Fourier difference techniques. All data sets were corrected for Lorentz and polarization effects. All structure solutions were performed using SHELXT.[28] All refinements were performed using SHELXL[29] via the Olex2 interface.[30]

Table S1. X-ray crystallographic data for 3.2a-b

Compound	**3a**	**3b**
Formula	$C_{27}H_{31}P$	$C_{26}H_{29}P$
$D_{calc.}$/ g cm^{-3}	1.186	1.205
μ/mm^{-1}	0.137	1.215
Formula Weight	386.49	372.46
Colour	colourless	colourless
Shape	blade	irregular-shaped
Size/mm^3	0.34×0.11×0.03	0.12×0.07×0.05
T/K	120(2)	100(2)
Crystal System	triclinic	monoclinic
Space Group	P-1	$P2_1/n$
a/Å	6.1700(12)	18.7310(10)
b/Å	10.939(2)	6.1796(3)
c/Å	16.649(4)	35.9587(19)
α/°	96.673(7)	90
β/°	94.197(7)	99.369(2)
γ/°	102.990(6)	90
V/Å^3	1081.8(4)	4106.7(4)
Z	2	8
Z'	1	2
Wavelength/Å	0.71073	1.54178
Radiation type	MoK_α	Cu K_α
Θ_{min}/°	1.929	2.491
Θ_{max}/°	25.434	75.114
Measured Refl's.	14647	80310
Indep't Refl's	3944	8333
Refl's I≥2 σ(I)	3056	6739
R_{int}	0.0581	0.0339
Parameters	256	491
Restraints	0	0
Largest Peak	0.246	0.867
Deepest Hole	-0.267	-0.368
GooF	1.020	1.056
wR_2 (all data)	0.1047	0.1101
wR_2	0.0964	0.1080
R_1 (all data)	0.0640	0.0543
R_1	0.0447	0.0466

Computational Details

All calculations were performed using version 5.0.1 of the ORCA computational package[31] and were run on the Cedar cluster maintained by Compute Canada. All geometry optimizations and frequency calculations were performed at the B3LYP-D3(BJ)/def2-TZVPP level of theory.[32-24] The RIJCOSX approximation was used to enhance computational efficiency, along with the auxiliary basis *def2/J*.[35] Convergence criteria were met using the *defgrid2* integral grid size. Frequency calculations were performed to confirm that each optimized geometry was a true minimum indicated by the absence of imaginary frequencies. Furthermore, the implicit solvation model CPCM (as integrated in ORCA) was applied with a dielectric constant of ε = 7.25 (corresponds to THF).[36]

Table S2. Energetic data (E_h = Hartrees) for solvated molecules calculated at B3LYP-D3(BJ)/def2-TZVPP level of theory with implicit solvation model (CPCM, ε = 7.25).

Compound	Total thermal energy in E_h	Total Enthalpy in E_h	Gibbs Free Energy at 298.15 K in E_h
**[2a]**⁻	-1349.574464	-1349.57352	-1349.654673
**3a**	-1388.194021	-1388.193077	-1388.273692
**[2a]***⁻	-1349.556776	-1349.555831	-1349.636397
**[4a]**⁻	-1889.963357	-1889.962412	-1890.065402
diphenylethylene	-540.3888994	-540.3879552	-540.4363912
diphenylmethanide	-501.7930074	-501.7920632	-501.838797

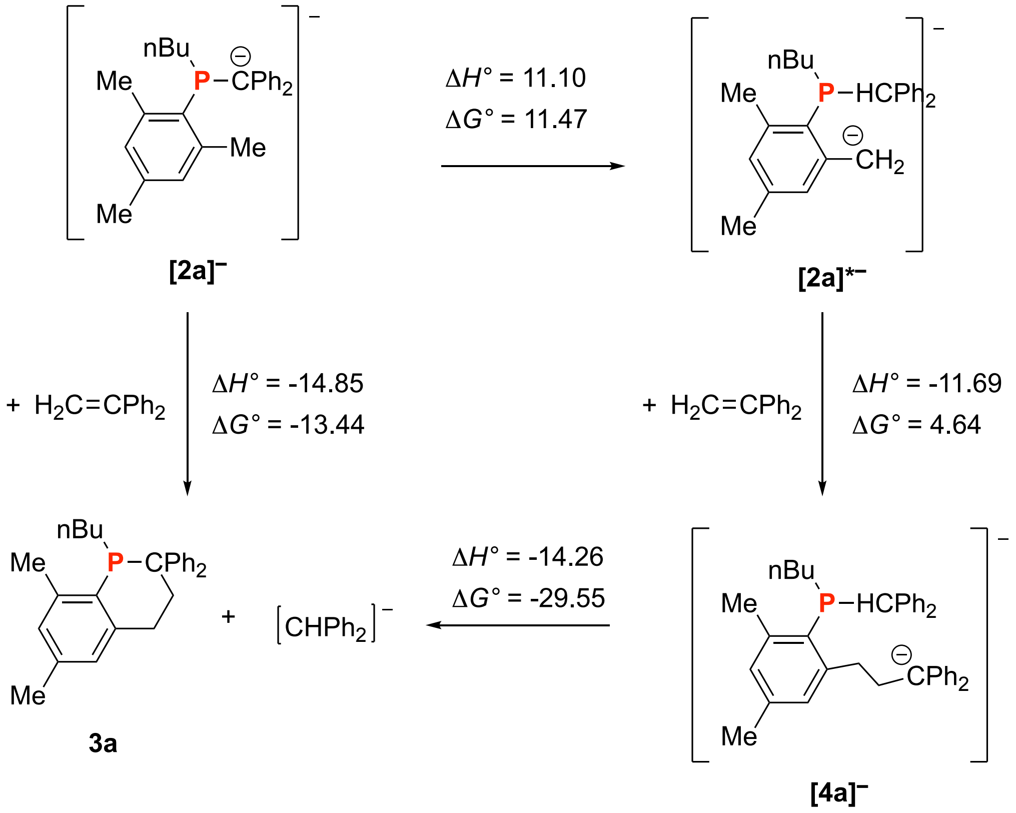

Scheme S1. Comparison of reaction enthalpies ΔH° and Gibbs free energies ΔG° (298.15 K) for cyclization reaction of [**2a**]⁻ and diphenylethylene including two intermediates. Energies are given in kcal mol^{-1}.

Table S3. Coordinates of DFT-optimized structures.

2a⁻

P	14.17026738760806	10.35520504339035	2.92530629778442
C	16.51740412739108	8.37087593097408	3.48818032234843
C	17.74861010474292	7.79794760427282	3.76414926916760
C	18.04031395706051	7.27993273785920	5.02768607657579
C	17.05247019559786	7.37024010665848	6.00877335024138
C	15.82116761885172	7.94696214867355	5.73752157586205
C	15.48336707080212	8.46988226711202	4.45906557916678
C	12.80979993408338	7.43860947650291	5.45988001595241
C	11.64399054301500	7.06998771122326	6.10800994566671
C	10.60607646622033	7.98582806305772	6.30572716685401
C	10.78432561173826	9.28229732160376	5.82786711091716
C	11.95385950792273	9.65550996391088	5.17958375612161
C	13.02456346289626	8.75154914324027	4.95532708713381
C	14.20699874493404	9.10548753296449	4.20203280822234
C	13.31396065133711	12.94725762833985	1.29482432215378
C	13.12428218212088	16.07532551461496	5.17501885443926
C	14.82993132513026	11.46141620075240	5.97499241308828
C	13.46152578288780	13.10855368397304	2.78968589761372
C	13.23034379453737	14.38991346151853	3.29567228373992
C	13.42919737522719	14.70517397540972	4.63185317735157
C	13.94300296850814	13.70105027721272	5.44616293657036
C	14.18821736308879	12.40666317481841	4.98759717376788
C	13.87286146933446	12.06139759665267	3.64832872328677
C	13.11934233950544	6.44646390159406	0.52265859154409
C	13.40005576211145	7.91330211559676	0.83875198237267
C	12.32563465804712	8.53569730871657	1.72828023026831
C	12.54425180585715	10.01729845342978	2.04107490617539
H	13.05968021025797	16.81247435569773	4.37426060652696
H	13.88805191524395	16.40223764669878	5.88259451035587
H	12.16868643659297	16.08276522233037	5.70680271782638
H	12.16201529205954	6.33072982295186	0.00803167205689
H	13.07411238812592	5.85505764637754	1.44007576016328

H	13.89519862834886	6.01589295780969	-0.11330666269863
H	13.48147935663148	13.90625494499028	0.80448435834905
H	12.31813540111082	12.60609517023225	1.01068708459765
H	14.03159342088261	12.23013023752144	0.89534022242455
H	15.14100728452052	12.02005904499957	6.85830510991695
H	15.70385609245525	10.96603804271476	5.55654937205654
H	14.15020595218128	10.67213676940982	6.28957920103040
H	14.36969354355300	7.99848223149724	1.33308604985306
H	13.47735400233136	8.48525363672290	-0.09190008658285
H	11.35498943644194	8.43318021376487	1.22899545106148
H	12.26013475324597	7.96775209539900	2.65626112412445
H	12.55398359902060	10.56388809327750	1.10173569661360
H	11.70525028928519	10.39935754914877	2.62299138584782
H	15.09715431252059	8.01914270451376	6.53668996363686
H	17.24932664907109	6.99840935964486	7.00827807010284
H	19.00040926975331	6.82833832146904	5.23961835004931
H	18.49059525146811	7.74346112688851	2.97520113816739
H	16.33048868899336	8.75598978111512	2.49567149085877
H	12.05105117219233	10.67510887757186	4.83775025548230
H	10.00316067407597	10.02116291888863	5.96817967023068
H	9.69498438623238	7.69629307218194	6.81246499235177
H	11.53396653448702	6.04808286825598	6.45347997546890
H	13.57450223366270	6.69072664937122	5.30248111112880
H	14.17786850373089	13.93193957674681	6.47945938776269
H	12.90513811096444	15.16588171773367	2.61094916684934

3a

P	1.27585432145328	5.48262987977910	4.56963411897188
C	-0.97449527420953	8.30287441421682	5.48465977854009
C	-0.89853337496822	9.37721967859072	6.36876048113393
C	0.27125658902314	10.11781577569539	6.47665370325537
C	1.36675834557496	9.78005228668201	5.68515272548832
C	1.28674830421625	8.71064542874901	4.80515784896748
C	0.11714305728944	7.94880270954722	4.69356765680918

C	0.01430750974472	8.25855495851244	1.74954971528763
C	0.39523233107700	8.66141344947157	0.47728094240948
C	1.40040159015493	7.97652420741595	-0.20192080875811
C	2.01213695317753	6.88971711625600	0.40827746757280
C	1.62315868113567	6.48832581456061	1.68479490415041
C	0.62115218819051	7.16506100683023	2.37630252798122
C	0.10831913942869	6.74270800625450	3.75948660091859
C	-1.28969876872567	6.11851511869208	3.59165356965153
C	-1.30301568823465	4.94429161523657	2.62593858977774
C	-0.35799806731523	0.06186199313703	2.12040447902937
C	2.74276633200447	2.81353164056354	4.93163451159256
C	-0.41220883984675	3.78050655971399	2.99366117368446
C	-0.73839904499869	2.53421029536642	2.44943249773537
C	0.01929957921137	1.39940280433276	2.69696683851127
C	1.14436854935571	1.53241719296134	3.51240088488983
C	1.50737788140661	2.75189466669783	4.06965126742320
C	0.71581362618381	3.89906531577787	3.81806030922723
C	3.24749476298484	6.63542035919185	8.76739724306545
C	2.63747016848186	5.76768712394196	7.67002651561942
C	1.18663287941385	6.13902104435649	7.37222352901098
C	0.53650065385818	5.28935374099787	6.27667393421530
H	-1.89524888385386	7.74245424284056	5.42649760410364
H	-1.75943878303798	9.63097654228327	6.97357784857622
H	0.33041032046719	10.94987396370228	7.16568371868154
H	2.28446295250979	10.34927802277725	5.75564599872519
H	2.14363622537313	8.45662701677567	4.19587868738326
H	-0.76139914072710	8.80592704205263	2.26900345678925
H	-0.08962682323471	9.51221975223079	0.01622236113628
H	1.70211394215724	8.28983590693647	-1.19267489898180
H	2.79481529533771	6.34675464943573	-0.10549025107025
H	2.11537635456839	5.63802836602805	2.13495430657931
H	-1.97938334391916	6.88286131661386	3.22622274826687
H	-1.67062291743624	5.79086643586765	4.55914384160651
H	-1.02118399373607	5.29626869114680	1.62923014690701
H	-2.32664341490947	4.57781434403241	2.53367852936230

H	-0.69584652446606	-0.62041477412455	2.90433277486788
H	-1.16169234139112	0.15815982453008	1.39117765265212
H	0.49528454476840	-0.41071160194291	1.63044881019642
H	2.49798329113717	2.99614608052052	5.97985096768517
H	3.29103212125742	1.87420630025668	4.87843869699667
H	3.40645555092863	3.62182663319485	4.62048109983315
H	-1.61545572274727	2.45772910447427	1.81642325673181
H	1.75845882911281	0.66207017839376	3.71391491817588
H	2.68794887916546	6.53844549811576	9.70079462125878
H	4.28364179920786	6.35811818036041	8.96850876678808
H	3.23235144498817	7.68968925284560	8.48136617903971
H	2.68715869647091	4.71361538344233	7.96011656788423
H	3.22958037965570	5.86180085376581	6.75650805488278
H	0.59762473566606	6.02267839379212	8.28774041281238
H	1.13218018295131	7.19599363424453	7.10939585057835
H	0.65026011273128	4.23186176020060	6.52088020976862
H	-0.53580315406369	5.47978780167706	6.23159305562033

[**2a**]$^{*-}$

P	14.35944508500286	10.44025537724699	3.09793781528402
C	13.14640093836818	9.51216106852867	2.04284097650528
C	13.68191887709380	8.14225456340257	1.59684271076109
C	14.91920914492692	8.17928882423794	0.70305377825659
C	15.39804235211578	6.78274781102529	0.31561047230719
C	13.70484008269050	12.04792232532521	3.62274207303108
C	14.72938488722248	12.87464400505142	4.27187316355656
C	14.33661050002849	14.22248992878317	4.60686787210507
C	13.07236807410435	14.70835825634370	4.41903362648905
C	12.08450465977903	13.85928044682157	3.87289710472519
C	12.39320840483450	12.55578010085720	3.49120620280089
C	16.01063402718802	12.45427431264308	4.60681702302319
C	12.71674679902784	16.12443373064774	4.79187539477937
C	11.23471253931637	11.73966682277544	2.96572614321621
C	14.31741999653104	9.46897043161499	4.76753891638320

C	12.92727900590575	9.41399754346632	5.31844959472774
C	12.51902445602596	10.41331146636523	6.20988681274690
C	11.21076888998005	10.48068566895718	6.67245612575967
C	10.27200364302895	9.54154117017062	6.25634453311024
C	10.66070114000408	8.53791732239011	5.37374270850815
C	11.96908521516611	8.47783102772304	4.90850889808406
C	15.12850970089254	8.19190725321361	4.71595799760567
C	14.69595649642109	6.97942495976737	5.25998051182377
C	15.48866236180628	5.83603992530278	5.20112666076826
C	16.74482714676841	5.87779437331204	4.60830200388100
C	17.20525322918474	7.08585699350171	4.09127739056327
C	16.41001249419068	8.22220969139384	4.14745303726901
H	14.88383897286835	10.17740829935990	5.37968980822763
H	11.07096520421783	14.22246697049223	3.75188215210133
H	15.09251288283799	14.86659483511841	5.04619535178310
H	12.24260900640770	7.69991203798623	4.21116774357144
H	9.94040523211387	7.80281750494697	5.03759164705483
H	9.25145444925996	9.59157239137498	6.61296095057089
H	10.92341580881805	11.27107139852250	7.35414119548193
H	13.24044637928286	11.15468898316154	6.52662166517055
H	16.77691744316190	9.14988302798863	3.73187693332593
H	18.18742641810146	7.14378157719963	3.63940800231861
H	17.35930280588463	4.98844565420436	4.55797763099764
H	15.11892474792496	4.91118474441976	5.62580371168612
H	13.73115004725919	6.91848655415208	5.73949007991010
H	12.18727297543847	9.37240761468244	2.53164158058328
H	12.97246557845220	10.13663976690273	1.16324525879655
H	13.89774518051093	7.52808258245136	2.47340307497645
H	12.87896931042187	7.62650374148702	1.05939458635269
H	14.69407015917177	8.75934922191670	-0.19769821818741
H	15.72173214433892	8.70973874352423	1.21699979734225
H	16.70572098564502	13.16405091929944	5.03934010155960
H	16.39943316095146	11.47998982198522	4.35691308358808
H	11.36636896893433	11.44338948597651	1.92559314300931
H	11.09098521285460	10.83178168557294	3.54948039041123

H	10.31698658878302	12.32379827421073	3.02821997619982
H	16.27826330945969	6.82588926276306	-0.32849319479764
H	15.66282030664965	6.20546522104013	1.20453954650893
H	14.61962954861366	6.23148530341415	-0.21793458337658
H	13.56454814275088	16.64287732186075	5.24080551305061
H	12.39588561137556	16.69592350995904	3.91627775564871
H	11.88781497570204	16.14884188185589	5.50482476596165

[**4a**]⁻

P	14.34073222240444	10.33547883084351	3.26013031189472
C	16.74819459827808	9.37100623415503	9.94322189276242
C	18.11352526117028	9.47014615236993	10.13597650196772
C	18.85105738727312	10.55377643361622	9.64958486581730
C	18.15130604742224	11.52349702168576	8.92885991039971
C	16.78500023566307	11.43285110454285	8.72248410997760
C	15.98310230739316	10.35954795584177	9.24023931611426
C	12.47995378320083	8.95946857969602	8.78294715074258
C	11.58762201374716	7.99811136102058	9.22556769194849
C	11.81581015769222	7.28119802186036	10.40193329817507
C	12.96909037263073	7.58836637736761	11.12807800870024
C	13.87063929548102	8.54440112500567	10.68967351950223
C	13.69066481596904	9.27626830756392	9.47566071847892
C	14.58337271206340	10.28118794562492	8.97599225852135
C	13.99473101579808	11.31588073547194	8.03564150091497
C	16.53114812738440	8.34913087717614	4.63529960357822
C	17.60288445392009	7.96931037322205	5.43395116779433
C	17.38486699495872	7.54592007657421	6.74221817034173
C	16.08702279873808	7.50372910444177	7.23312241607124
C	15.01757222150587	7.88755608332477	6.43325471886726
C	15.22205587850771	8.32393199362235	5.12633652731011
C	12.35612176360734	6.99698864516224	4.78129945921323
C	11.11027385340178	6.58133335813912	5.23018033091960
C	10.15704017711392	7.52248780872679	5.61005054020260
C	10.46655090763132	8.87355704281507	5.52839550484436

C	11.72011475907779	9.28316117997269	5.08239744168556
C	12.68891607555539	8.35399762044086	4.71089066151328
C	14.07343310734212	8.71836226385338	4.20616847421347
C	14.12946374936979	13.06836738740163	1.84912611368953
C	12.66344305450187	15.73879710403032	5.79070475875162
C	14.21181207191306	11.02911010401736	6.54142146511291
C	13.78391989010009	13.04957036831243	3.32224183564763
C	13.39175200005482	14.27463934116246	3.86715205068873
C	13.14043186581944	14.43142755483788	5.21955222415556
C	13.38383572142781	13.33675313315429	6.04539090267489
C	13.79563781755922	12.09911179696132	5.55756042132412
C	13.89959488788612	11.91030344617337	4.15399033516003
C	13.92132781379015	7.35710400161269	-0.41945065926244
C	14.05354468588042	8.61727235456455	0.43158582154645
C	12.81894608276112	8.87080197065209	1.29455527018763
C	12.86525555532170	10.16452534993717	2.11452387288837
H	16.26157236601711	8.48010549601815	10.30090938126472
H	18.61992206885452	8.67127867463290	10.66763178766255
H	19.91850187097336	10.62798332174084	9.80843181995453
H	18.68414460893165	12.36977312838010	8.50808847823653
H	16.32257360878330	12.20849069148565	8.13096411321768
H	14.22402660113435	8.07536543245299	3.33238455991403
H	12.25482184396225	9.45835008942709	7.85301699211093
H	10.70498787867273	7.79588511535599	8.62949759245832
H	11.12199294796177	6.52439351859985	10.74310596482946
H	13.16296241397164	7.08262280779483	12.06798501796335
H	14.70569880857781	8.77788577478218	11.33066438926040
H	14.41359525610531	12.29434383780415	8.27750997572740
H	12.92190334516848	11.41455036379956	8.21795879582538
H	13.70514612664767	10.10099838029452	6.30882152487090
H	15.27178526052753	10.82008628889452	6.37885994262820
H	12.76998687398973	16.54876933241019	5.06921663405716
H	13.21998439063849	16.00377760001458	6.69126901634235
H	11.60853821156908	15.67976273760213	6.07188800498083
H	13.06528209925799	7.42717931103070	-1.09475477790705

H	13.77396809633224	6.47670988431590	0.21036749267820
H	14.81290356579991	7.18831464708731	-1.02561789138723
H	14.59805032113200	14.02173459220784	1.60422512765115
H	13.24574967455530	12.97535967180771	1.21592013226452
H	14.82093384331965	12.27361802280639	1.57269728455948
H	14.93809047107788	8.53495459169152	1.06841205179853
H	14.22483775220119	9.48538635758944	-0.21209343026270
H	11.94133892795743	8.92611931818558	0.64233669249950
H	12.64656152152992	8.01086421742405	1.94516599571234
H	12.90246936162185	11.00144723539834	1.42455399867112
H	11.94668976330707	10.27005175441820	2.69004271062419
H	14.02198350084340	7.86350200566905	6.84951131781370
H	15.90051885035358	7.20190041450822	8.25351087836260
H	18.21736167431957	7.26664302342437	7.37413546443995
H	18.60816184928562	8.00500753363628	5.03368722304491
H	16.71100737976426	8.67672952822375	3.61979401240688
H	11.93403639731491	10.33925444465549	5.02435716305549
H	9.73538957151756	9.61725865919917	5.81707533792054
H	9.18551823897948	7.20406314382412	5.96413912178797
H	10.88322137391246	5.52445631936465	5.28363095583964
H	13.09245353415478	6.25867425921129	4.48857277413344
H	13.27020170386779	13.46760381544970	7.11107160856238
H	13.30098459028672	15.12828221602302	3.20475738548659

diphenylethylene

C	-1.31155465917215	1.63480375589790	-1.71104317925383
C	-0.69934492983001	2.33377064119507	-2.74355887495811
C	-0.82717695461805	3.71746005008058	-2.82474700001499
C	-1.57329585466769	4.39449026142803	-1.86518771088994
C	-2.19065335279772	3.69326349642669	-0.83630864293859
C	-2.07613966418981	2.30202769702981	-0.74727395411883
C	-0.67809186151282	0.51699288296668	1.25062335061743
C	-0.00551217136178	-0.51770732171491	1.88814575887142
C	-0.68268645619452	-1.68165827095495	2.23999236161334

C	-2.03824163381707	-1.80021556116834	1.94934881939105
C	-2.71121207803625	-0.76212913610193	1.31639215465241
C	-2.04439954931170	0.41608975654573	0.96406287413044
C	-2.76832753399416	1.54480305624731	0.32611041133171
C	-4.00925238642975	1.87266414933356	0.70813616716006
H	-1.20603193764816	0.55987215564185	-1.65546807963048
H	-0.12156916728658	1.79776394721388	-3.48514730070678
H	-0.34388099832536	4.26335117562550	-3.62418266226741
H	-1.66746065984171	5.47160037731595	-1.91172119621973
H	-2.75396473328240	4.22799030836699	-0.08355053293581
H	-0.14288600165995	1.41728093192622	0.98118340046888
H	1.04868226442935	-0.41498693159579	2.11007704867707
H	-0.15736062207257	-2.49105922359058	2.72938504801871
H	-2.57093703071356	-2.70646544222844	2.20651159675056
H	-3.76040095822879	-0.86983353940146	1.07660758780398
H	-4.55572644068192	2.66898021698447	0.22184283135255
H	-4.50683180055484	1.35249801393012	1.51499696059491

diphenylmethanide

C	-2.14159707222973	0.72425006006975	-0.50765235727664
C	-1.72521655238084	0.16023320851055	0.72593804732840
C	-2.41733733037460	-0.99729085986594	1.21086361154949
C	-2.09241625396323	-1.62770342385048	2.39394424675496
C	-1.04316506364843	-1.16191421274717	3.19910484350733
C	-0.33805496042528	-0.04511596433911	2.75406295991389
C	-0.65617932722219	0.60020555292526	1.56593829921440
C	-1.73626730877388	1.87897545982539	-1.22582932879152
C	-0.80652751720376	2.88092794397405	-0.80888928362341
C	-0.49568794359895	3.97518632643367	-1.60591059156760
C	-1.07267016686704	4.16255520186569	-2.86048935287364
C	-1.99893488761277	3.20441770938958	-3.29648176912432
C	-2.31755289248923	2.11398214533798	-2.51443207055551
H	-2.92635303561795	0.15827012702761	-1.00217612892249
H	-3.23487834923987	-1.38638928948606	0.61375204264845

H	-2.66169297418601	-2.49813946763430	2.70142365303389
H	-0.78838450025320	-1.65350124038916	4.12837333425198
H	0.48857403864448	0.33600384681970	3.34417157337955
H	-0.04775831487963	1.44005910129428	1.27569969083598
H	-0.33893649465250	2.82056848020069	0.15948656013373
H	0.21552053315181	4.70313652452190	-1.23035804228414
H	-0.81993828307480	5.01734024805271	-3.47330406922598
H	-2.47653129781935	3.31689690074342	-4.26371462681523
H	-3.03841477818303	1.39264858451997	-2.88389701169155

NMR Spectra of 3a, 3b and stack plots of reaction mixture

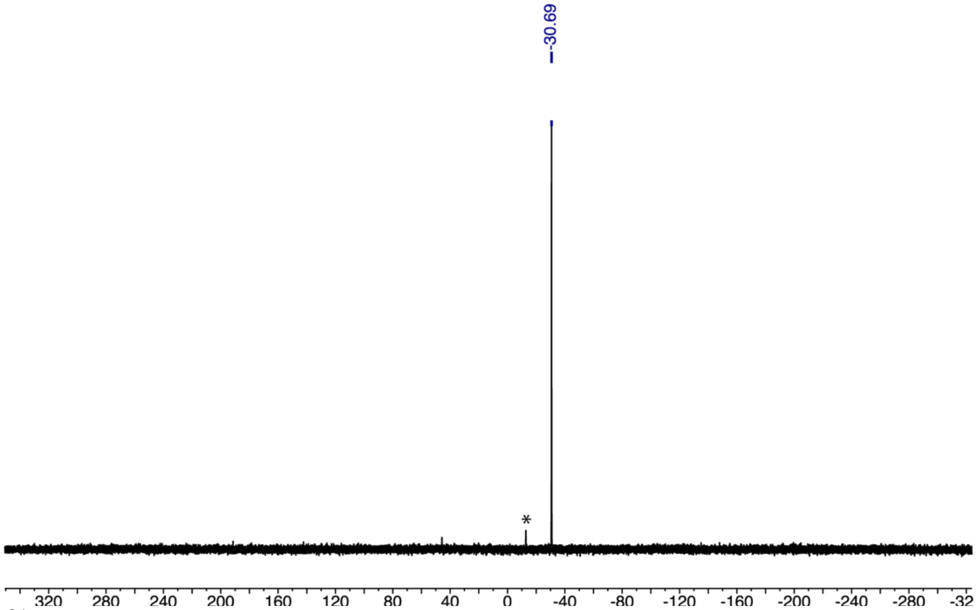

Figure S1 ^{31}P NMR spectrum (162 MHz, $CDCl_3$) of **3a.** * denotes signals assigned to quenched product, ArP(Bu)–CHR_2, presumably formed from traces protic impurities

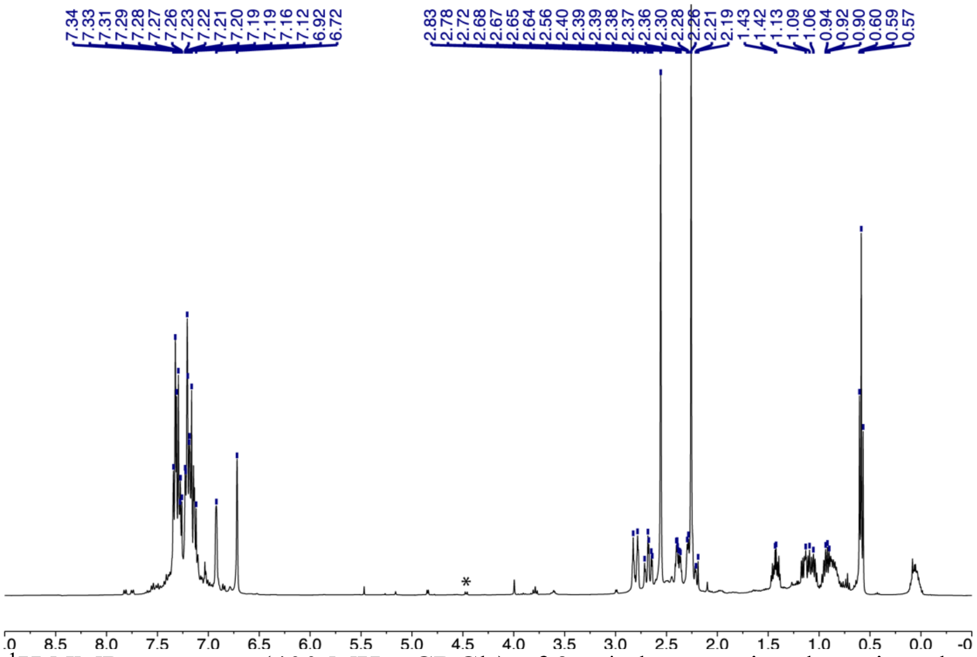

Figure S2 ^{1}H NMR spectrum (400 MHz, $CDCl_3$) of **3a**. * denotes signals assigned to quenched product, ArP(Bu)–CHR_2, presumably formed from traces protic impurities.

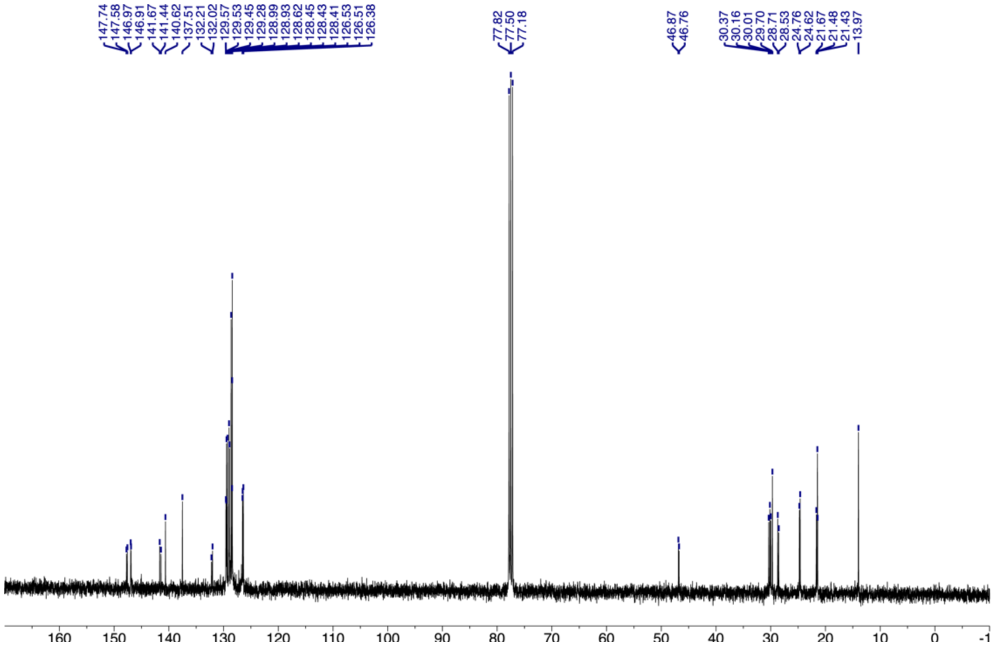

Figure S3 ^{13}C NMR spectrum (101 MHz, $CDCl_3$) of **3a**

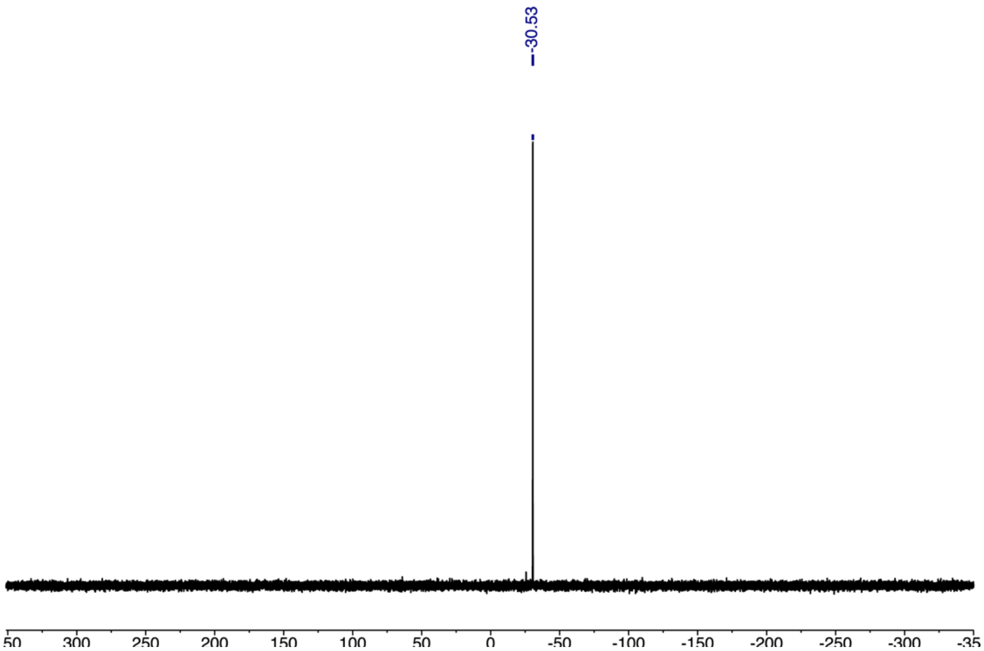

Figure S4 ^{31}P NMR spectrum (162 MHz, $CDCl_3$) of **3b**

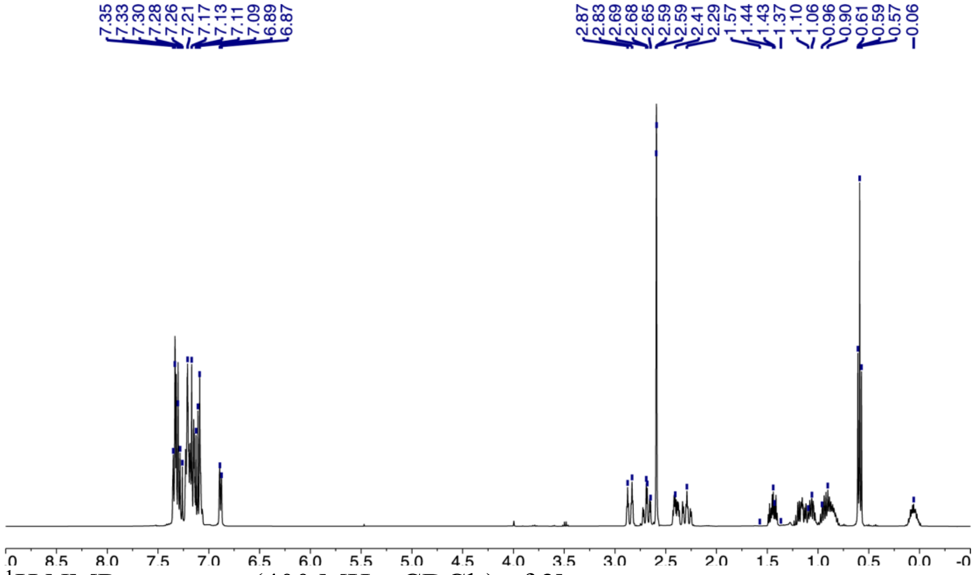

Figure S5 ^{1}H NMR spectrum (400 MHz, $CDCl_3$) of **3b**

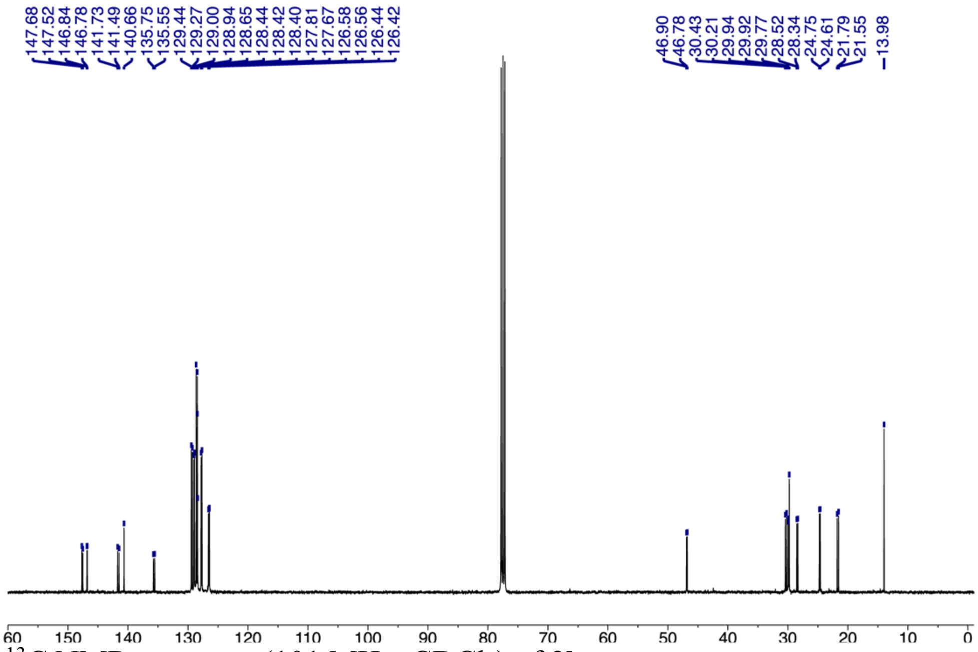

Figure S6 ^{13}C NMR spectrum (101 MHz, $CDCl_3$) of **3b**

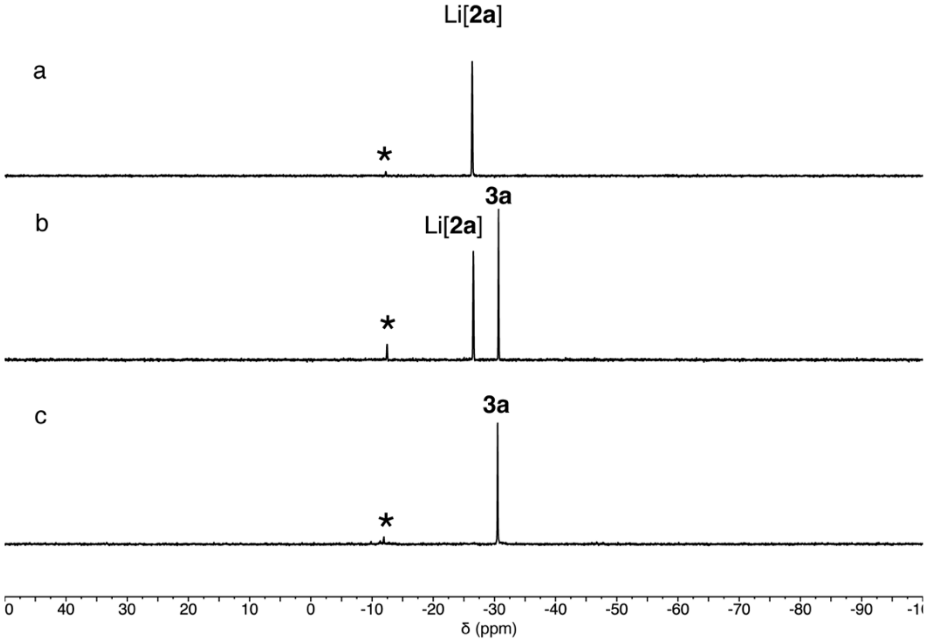

Figure S7 ^{31}P NMR (162 MHz, THF, 198 K) spectra of progress of Li[**2a**] with H_2C=CPh_2 (1 equiv) to afford cyclization product **3a**: a) solutions of Li[**2a**] in THF; b) immediately after addition of H_2C=CPh_2 (1 equiv); c) one day after addition of H_2C=CPh_2 (1 equiv). * denotes signals assigned to quenched product, ArP(Bu)–CHR_2, presumably formed from trace protic impurities.

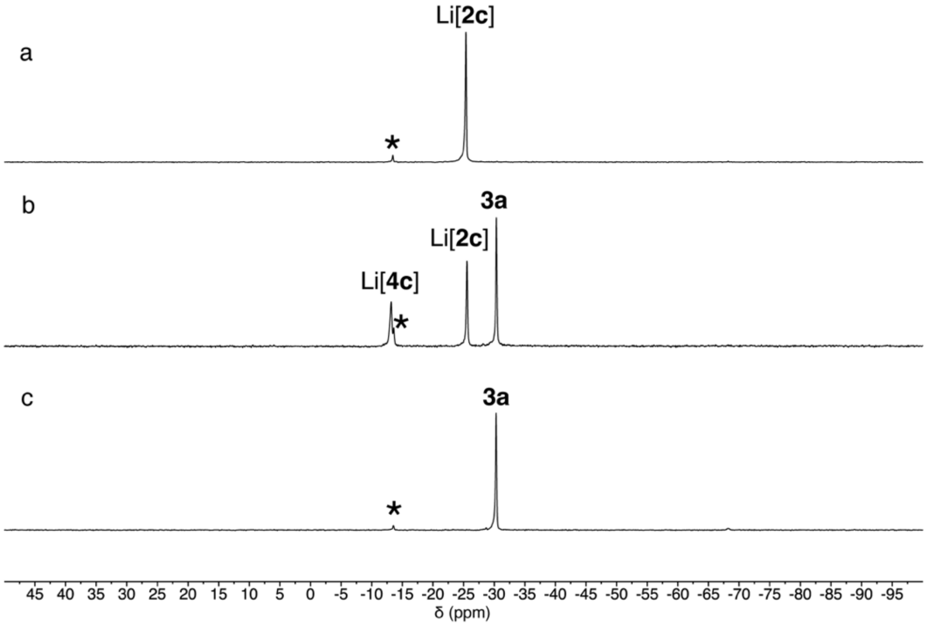

Figure S8 ^{31}P NMR (162 MHz, THF, 198 K) spectra of progress of Li[**2c**] with $H_2C{=}CPh_2$ (1 equiv) to afford cyclization product **3a**: a) solutions of Li[**2c**] in THF; b) immediately after addition of $H_2C{=}CPh_2$ (1 equiv); c) one day after addition of $H_2C{=}CPh_2$ (1 equiv). * denotes signals assigned to quenched product, ArP(Bu)–CHR_2, presumably formed from trace protic impurities.

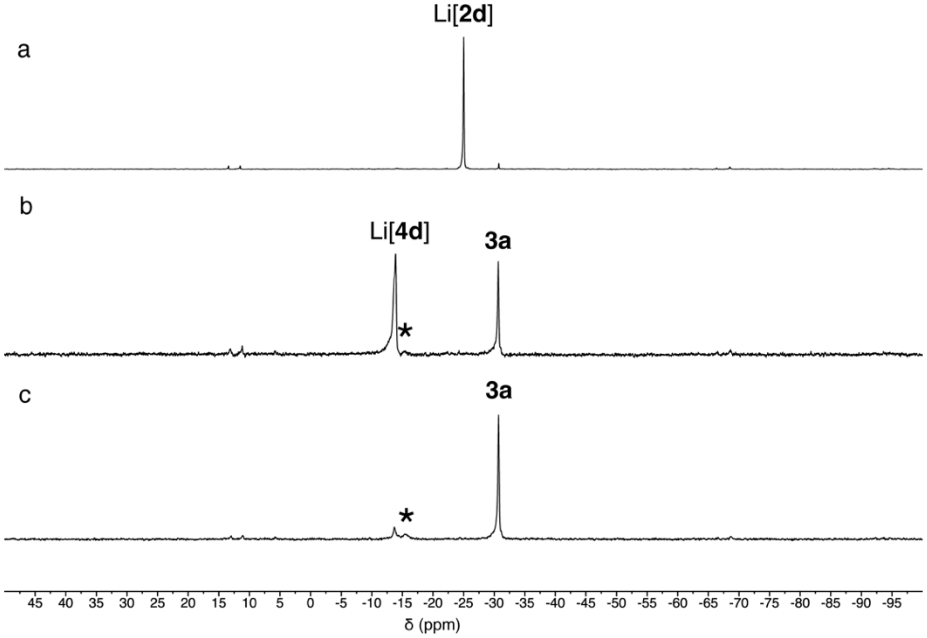

Figure S9 ^{31}P NMR (162 MHz, THF, 198 K) spectra of progress of Li[**2d**] with $H_2C{=}CPh_2$ (1 equiv) to afford cyclization product **3a**: a) solutions of Li[**2d**] in THF; b) immediately after addition of $H_2C{=}CPh_2$ (1 equiv); c) one day after addition of $H_2C{=}CPh_2$ (1 equiv). * denotes signals assigned to quenched product, ArP(Bu)–CHR_2, presumably formed from trace protic impurities.

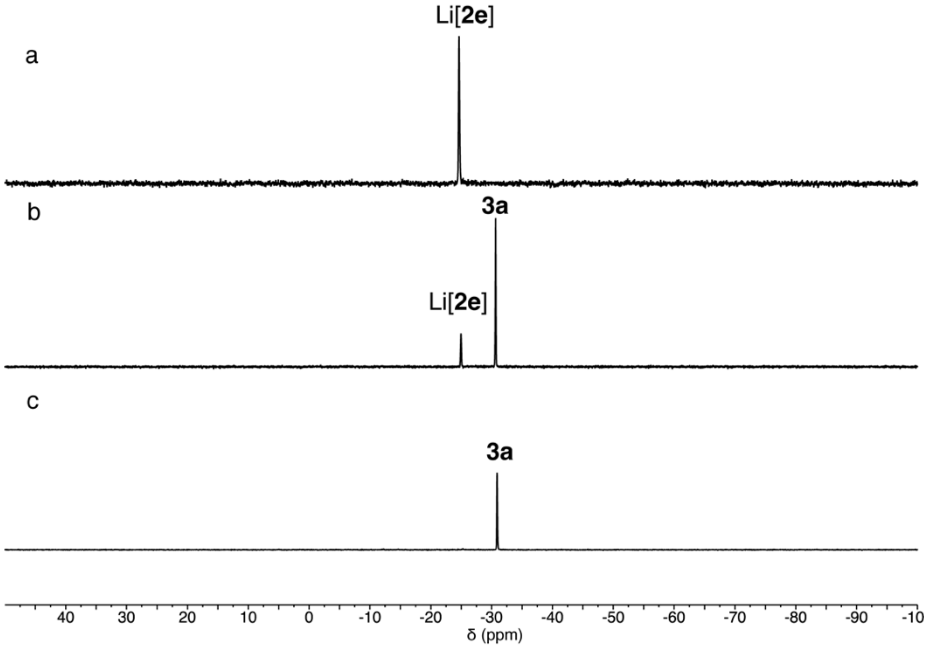

Figure S10 ^{31}P NMR (162 MHz, THF, 198 K) spectra of progress of Li[**2e**] with H_2C=CPh_2 (1 equiv) to afford cyclization product **3a**: a) solutions of Li[**2e**] in THF; b) immediately after addition of H_2C=CPh_2 (1 equiv); c) one day after addition of H_2C=CPh_2 (1 equiv). * denotes signals assigned to quenched product, ArP(Bu)–CHR_2, presumably formed from trace protic impurities.

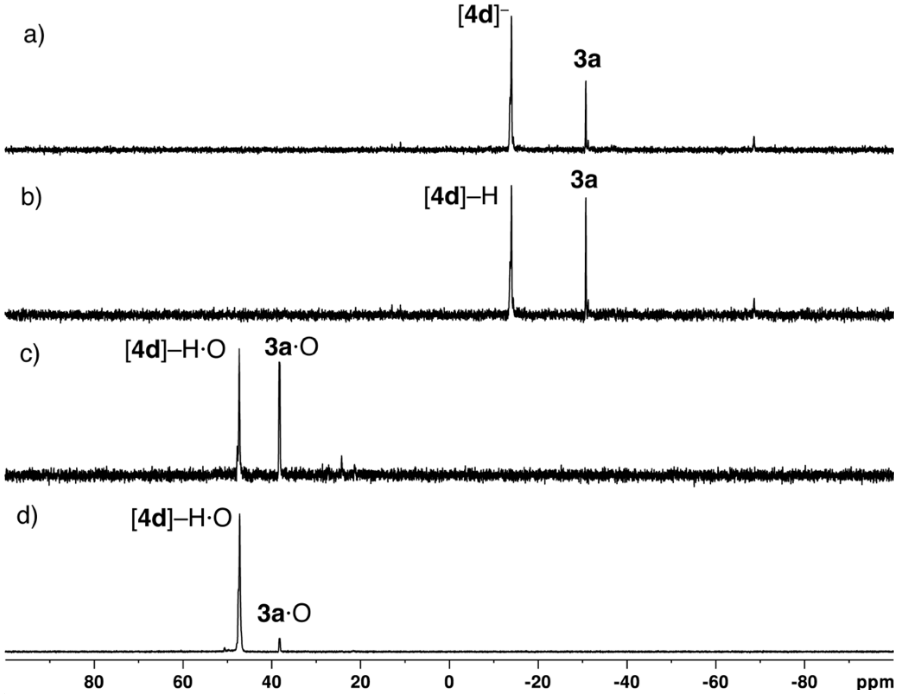

Figure S11 ^{31}P NMR (162 MHz, THF, 198 K) spectra showing the procedure used to identify intermediate, Li[**4d**] in the reaction of Li[**2d**] with H_2C=CPh_2 (1 equiv) to afford **3a** and $LiCHPh_2$: a) reaction mixture immediately after adding CH_2C=CPh_2 to Li[**2d**] in THF; b) reaction mixture immediately after quenching with one drop of methanol; c) prodiuct mixture immediately after

oxidation of the with H_2O_2 in CH_2Cl_2; d) recovered solid after column chromatography column (stationary phase: silica; mobile phase: CH_2Cl_2).

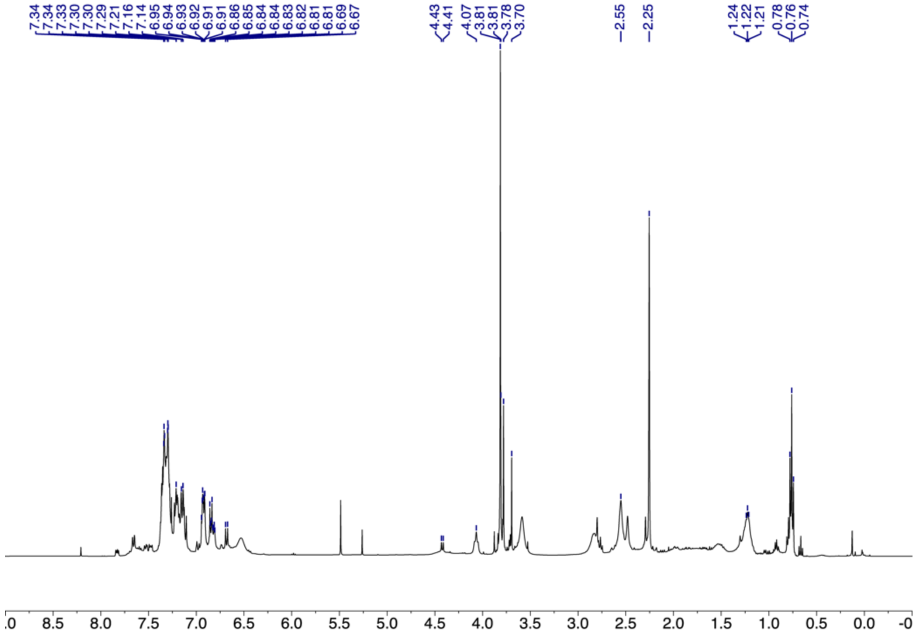

Figure S12 ^{1}H NMR spectrum (400 MHz, $CDCl_3$) of **[4d]–H·O**.

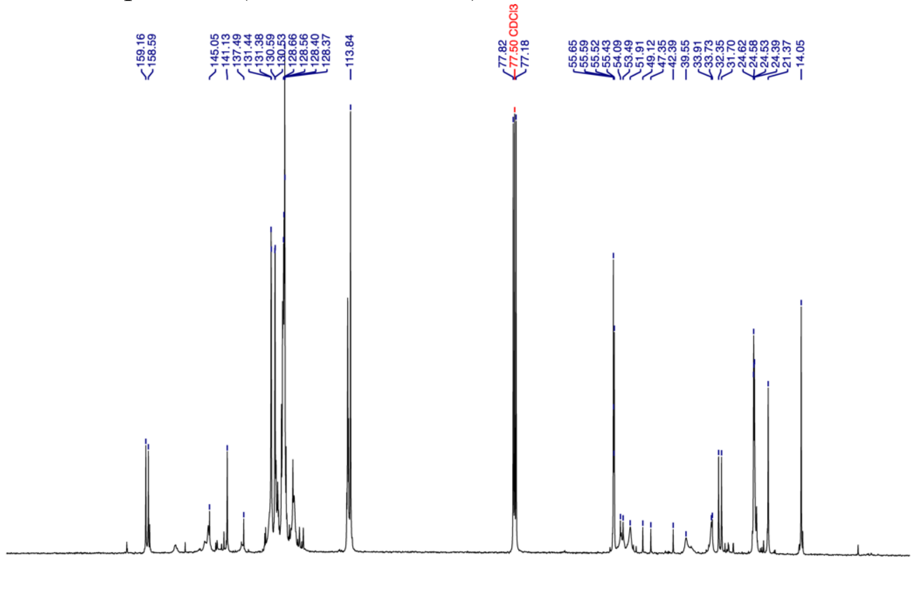

Figure S13 ^{13}C NMR spectrum (101 MHz, $CDCl_3$) of **[4d]–H·O**.

[25] N. R. Babij, E. O. McCusker, G. T. Whiteker, B. Canturk, N. Choy, L. C. Creemer, C. V. D. Amicis, N. M. Hewlett, P. L. Johnson, J. A. Knobelsdorf, F. Li, B. A. Lorsbach, B. M. Nugent, S. J. Ryan, M. R. Smith, Q. Yang, *Org. Process Res. Dev.* **2016**, *20*, 661.
[26] G. R. Fulmer, A. J. M. Miller, N. H. Sherden, H. E. Gottlieb, A. Nudelman, B. M. Stoltz, J. E. Bercaw, K. I. Goldberg, *Organometallics* **2010**, *29*, 2176.
[27] v. A. B. A. I. M. SAINT, WI 2013.
[28] G. M. Sheldrick, *Acta Crystallogr. A: Found. Adv.* **2015**, *71*, 3.
[29] G. M. Sheldrick, *Acta crystallogr., C Struct. chem.* **2015**, *71*, 3.
[30] O. V. Dolomanov, L. J. Bourhis, R. J. Gildea, J. A. K. Howard, H. Puschmann, *J. Appl. Crystallogr.* **2009**, *42*, 339.
[31] F. Neese, *Wiley Interdiscip. Rev. Comput. Mol. Sci.* **2022**, *12*, e1606.
[32] S. Grimme, J. Antony, S. Ehrlich, H. Krieg, *J. Chem. Phys.* **2010**, *132*.
[33] S. Grimme, S. Ehrlich, L. Goerigk, *J. Comput. Chem.* **2011**, *32*, 1456.
[34] F. Weigend, R. Ahlrichs, *Phys. Chem. Chem. Phys.* **2005**, *7*, 3297.
[35] F. Weigend, *Phys. Chem. Chem. Phys.* **2006**, *8*, 1057.
[36] V. Barone, M. Cossi, *J. Phys. Chem. A* **1998**, *102*, 1995.
